# Homocysteine metabolites inhibit autophagy by upregulating miR-21-5p, miR-155-5p, miR-216-5p, and miR-320c-3p in human vascular endothelial cells

**DOI:** 10.1038/s41598-024-57750-3

**Published:** 2024-03-26

**Authors:** Łukasz Witucki, Hieronim Jakubowski

**Affiliations:** 1 Department of Biochemistry and Biotechnology, Poznań University of Life Sciences, 60-632 Poznań, Poland; 2 Department of Microbiology, Biochemistry and Molecular Genetics, New Jersey Medical School, International Center for Public Health, Rutgers University, 225 Warren Street, Newark, NJ 07103 USA

**Keywords:** Homocysteine metabolites, microRNA, Autophagy, Endothelial dysfunction, HUVEC, Proteins, RNA, Non-coding RNAs, Biochemistry, Molecular biology

## Abstract

Nutritional and genetic deficiencies in homocysteine (Hcy) metabolism lead to hyperhomocysteinemia (HHcy) and cause endothelial dysfunction, a hallmark of atherosclerosis, which is a major cause of cardiovascular disease (CVD). Impaired autophagy causes the accumulation of damaged proteins and organelles and is associated with CVD. Biochemically, HHcy is characterized by elevated levels of Hcy and its metabolites, Hcy-thiolactone and *N*-Hcy-protein. However, whether these metabolites can dysregulate mTOR signaling and autophagy in endothelial cells is not known. Here, we examined the influence of Hcy-thiolactone, *N*-Hcy-protein, and Hcy on autophagy human umbilical vein endothelial cells. We found that treatments with Hcy-thiolactone, *N*-Hcy-protein, or Hcy significantly downregulated beclin 1 (BECN1), autophagy-related 5 (ATG5), autophagy-related 7 (ATG7), and microtubule-associated protein 1 light chain 3 (LC3) mRNA and protein levels. We also found that these changes were mediated by upregulation by Hcy-thiolactone, *N*-Hcy-protein, and Hcy of autophagy-targeting microRNA (miR): miR-21, miR-155, miR-216, and miR-320c. The effects of these metabolites on levels of miR targeting autophagy as well as on the levels of BECN1, ATG5, ATG7, and LC3 mRNA and protein were abrogated by treatments with inhibitors of miR-21, miR-155, miR-216, and mir320c. Taken together, our findings show that Hcy metabolites can upregulate miR-21, miR-155, miR-216, and mir320c, which then downregulate autophagy in human endothelial cells, important for vascular homeostasis.

## Introduction

Atherosclerosis is an inflammatory disease^1,2^ that underlies various manifestation of cardiovascular disease (CVD) such as myocardial infarction, stroke, and peripheral artery disease, the leading cause of mortality and morbidity. Endothelial cells form a monolayer on the luminal surface of arteries and veins that regulates the vascular tone and permeability, thereby supporting vascular homeostasis. Endothelial activation/dysfunction, the first step in atherosclerosis, is caused by biochemical or mechanical factors which disrupt vascular homeostasis and induce inflammation. In addition to elevated low-density lipoprotein, cigarette smoking, hypertension, diabetes mellitus, infectious microorganisms, and genetic alterations, endothelial dysfunction can be caused by elevated plasma homocysteine (Hcy), i.e., hyperhomocysteinemia (HHcy)^2^. Endothelial dysfunction and inflammation are associated with HHcy in animal models and humans^3–5^. Treatments of human umbilical vein endothelial cells (HUVEC) with Hcy also lead to endothelial activation/dysfunction manifested by induction of VCAM-1 expression without affecting cell viability^6^.

In addition to elevated Hcy, HHcy is characterized by elevated levels of Hcy-thiolactone and *N*-Hcy-protein, observed in nutritional or genetic deficiencies in Hcy metabolism in animals and humans^7^. HHcy is also characterized by pro-atherogenic changes in gene expression in mice and humans^8,9^.

HUVEC can metabolize Hcy to Hcy-thiolactone and *N*-Hcy-protein^10^. The levels of Hcy-thiolactone and *N*-Hcy are regulated by extracellular Hcy, folic acid, and HDL, factors that determine the susceptibility to CVD in humans, suggesting that Hcy-thiolactone and *N*-Hcy-protein could be involved in endothelial dysfunction and atherosclerosis^10^. Further, Hcy-thiolactone and *N*-Hcy can induce pro‑atherogenic patterns of gene expression, with Hcy-thiolactone upregulating LAMTOR2 mRNA, a component of the mammalian target of rapamycin (mTOR) signaling pathway in HUVEC^11^. However, mechanisms by which these metabolites can affect gene expression in vascular endothelial cells are not known.

MicroRNAs (miRs) are small non-coding RNAs playing a crucial role in cell physiology by regulating gene expression at the mRNA level^12^. miR genes are transcribed by RNA polymerase II to primary miR, which is first cleaved by DROSHA and then by DICER^13,14^, generating 21–23 nt double strand mature miRs^15^. One strand is incorporated into RISC (RNA-induced silencing complex) and regulates gene expression by binding to partially complementary mRNA sequence (mainly found in 3′ untranslated region (3′ UTR)) to induce translational repression, mRNA deadenylation or cleavage^16^. Dysregulated miR expression can lead to endothelial dysfunction^17^, CVD^18^, stroke^19^, and neuropathies^20,21^.

Autophagy is an evolutionarily conserved cellular process involving degradation and recycling damaged proteins and organelles. Autophagy occurs continually at basal levels in cells and contributes to the maintenance of cellular homeostasis. Impaired autophagy causes the accumulation of damaged proteins and organelles^22^ and is associated with CVD^23–25^.

Our earlier studies demonstrated that HHcy resulted in impaired autophagy in brains of mice with genetic deficiencies in the metabolism of Hcy (*Cbs*^−/−^ mice^26,27^) and Hcy-thiolactone (*Blmh*^−/−^ mice^28^; *Pon1*^−/−^ mice^29^), and that specific metabolites associated with HHcy such as *N*-Hcy-protein, Hcy-thiolactone, and Hcy can impair autophagy in cultured mouse neuroblastoma cells^27–29^. However, whether miRs can mediate the effects of these metabolites on autophagy were not known.

We hypothesize that HHcy-associated metabolites affect the expression of autophagy-related genes via a miR-mediated mechanism in human endothelial cells. To evaluate this hypothesis, we studied the influence of *N*-Hcy-protein, Hcy-thiolactone, and Hcy on autophagy-related protein and mRNA levels as well as on levels of miRs targeting autophagy-related mRNAs in HUVEC. We also studied how miR inhibitors affect the expression of autophagy-related genes.

## Results

### Hcy-thiolactone, *N*-Hcy-protein, and Hcy downregulate autophagy-related proteins

**Figure 1:**
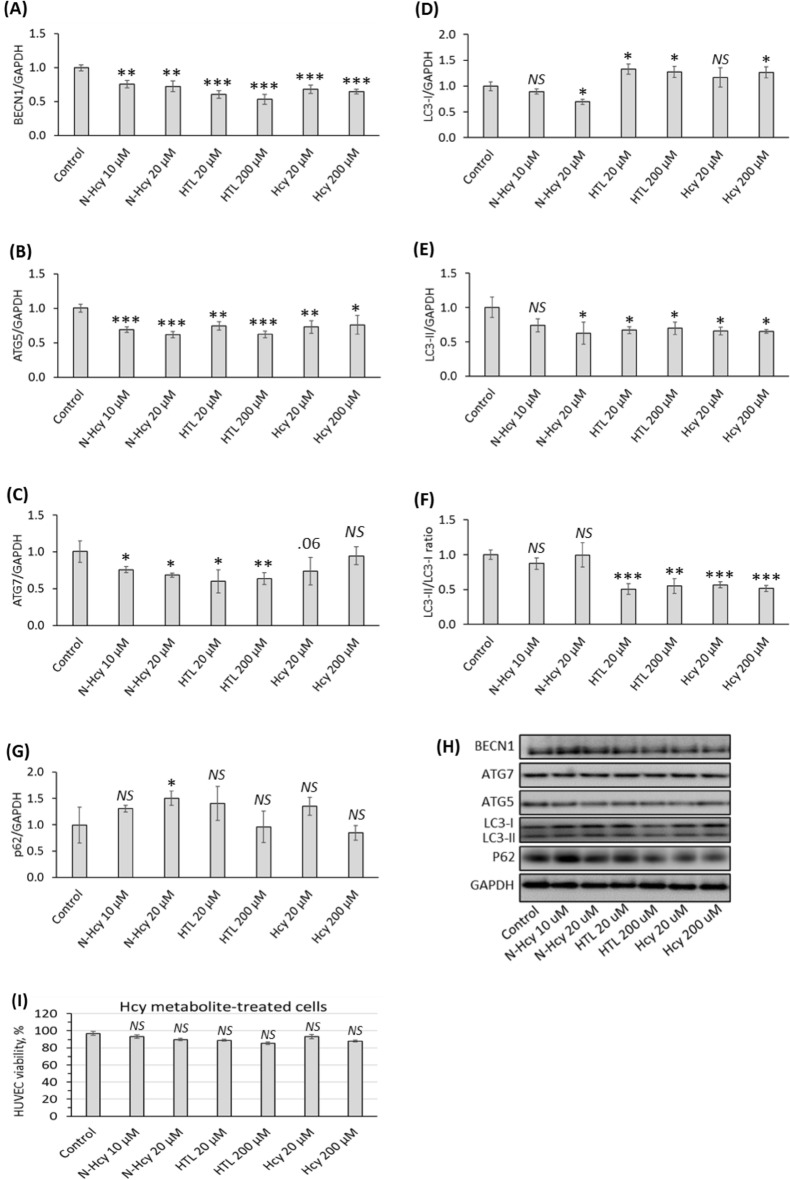
Influence of *N*-Hcy-protein, Hcy-thiolactone, and Hcy on the expression of autophagy-related proteins in HUVEC. Bar graphs show levels of BECN1 protein (**A**), ATG5 protein (**B**), ATG7 protein (**C**), LC3-I (**D**), LC3-II (**E**), LC3-II/LC3-I ratio (**F**), and p62 protein (**G**) in HUVEC treated with *N*-Hcy-protein (N-Hcy), Hcy-thiolactone (HTL), or Hcy in methionine-free M199/dialyzed FBS medium for 24 h. Showed proteins were quantified by Western blotting. GAPDH was used as reference for the quantification of other proteins. Panel (**H**) shows representative images of Western blots. The treatments with Hcy, N-Hcy, and HTL did not affect HUVEC viability (**I**). Each assay was repeated three times (technical repeats) in three independent experiments (biological repeats). Mean ± SD values of three biological repeats for each treatment group are shown. *P < 0.05, **P < 0.01, ***P < 0.001 from one-way ANOVA with Tukey’s multiple comparisons test.

In an earlier work, one of us (HJ) has shown that HUVEC can metabolize Hcy to Hcy-thiolactone and *N*-Hcy-protein^10^. We have also shown that these metabolites reduced autophagy flux in mouse neuroblastoma N2A cells^28^. To figure out whether each of these metabolites can affect autophagy in human endothelial cells, we treated HUVECs with *N*-Hcy-protein, Hcy-thiolactone, or Hcy and quantified levels of autophagy-related proteins by Western blotting. We found significantly attenuated levels of regulators of autophagosome assembly beclin 1 (BECN1) (Fig. 1A) and autophagy-related 5 (ATG5) (Fig. 1B), in HUVEC treated with *N*-Hcy-protein, Hcy-thiolactone, or Hcy, compared with untreated controls. Autophagy-related 7 (ATG7) was significantly reduced in HUVEC treated with *N*-Hcy-protein or Hcy-thiolactone, but not with Hcy (Fig. 1C). Levels of microtubule-associated protein 1 light chain 3 (LC3-I), were significantly upregulated by Hcy-thiolactone and Hcy and downregulated by *N*-Hcy-protein (Fig. 1D). In contrast, the lipidated LC3-II was significantly downregulated (Fig. 1E). The LC3-II/LC3-I ratio, an indicator of autophagy flux, was reduced by Hcy-thiolactone and Hcy but unaffected by *N*-Hcy-protein (Fig. 1F). Protein p62, a receptor for the degradation of ubiquitinated substrates, known to be negatively correlated with autophagy flux^30^, was upregulated by *N*-Hcy-protein but unaffected by Hcy-thiolactone and Hcy (Fig. 1G). Representative images of western blots are shown in Fig. 1H. Cell viability was not significantly affected in HUVEC treated with *N*-Hcy-protein, Hcy-thiolactone, or Hcy, compared to untreated control (Fig. 1I), consistent with previous findings with Hcy-treated HUVEC^6^.

These findings show that Hcy-thiolactone and Hcy impair autophagy by dysregulating autophagosome assembly and autophagy flux while *N*-Hcy-protein dysregulates autophagosome assembly but has no effect on autophagy flux.

### Hcy-thiolactone, *N*-Hcy-protein, and Hcy downregulate autophagy-related mRNAs

**Figure 2:**
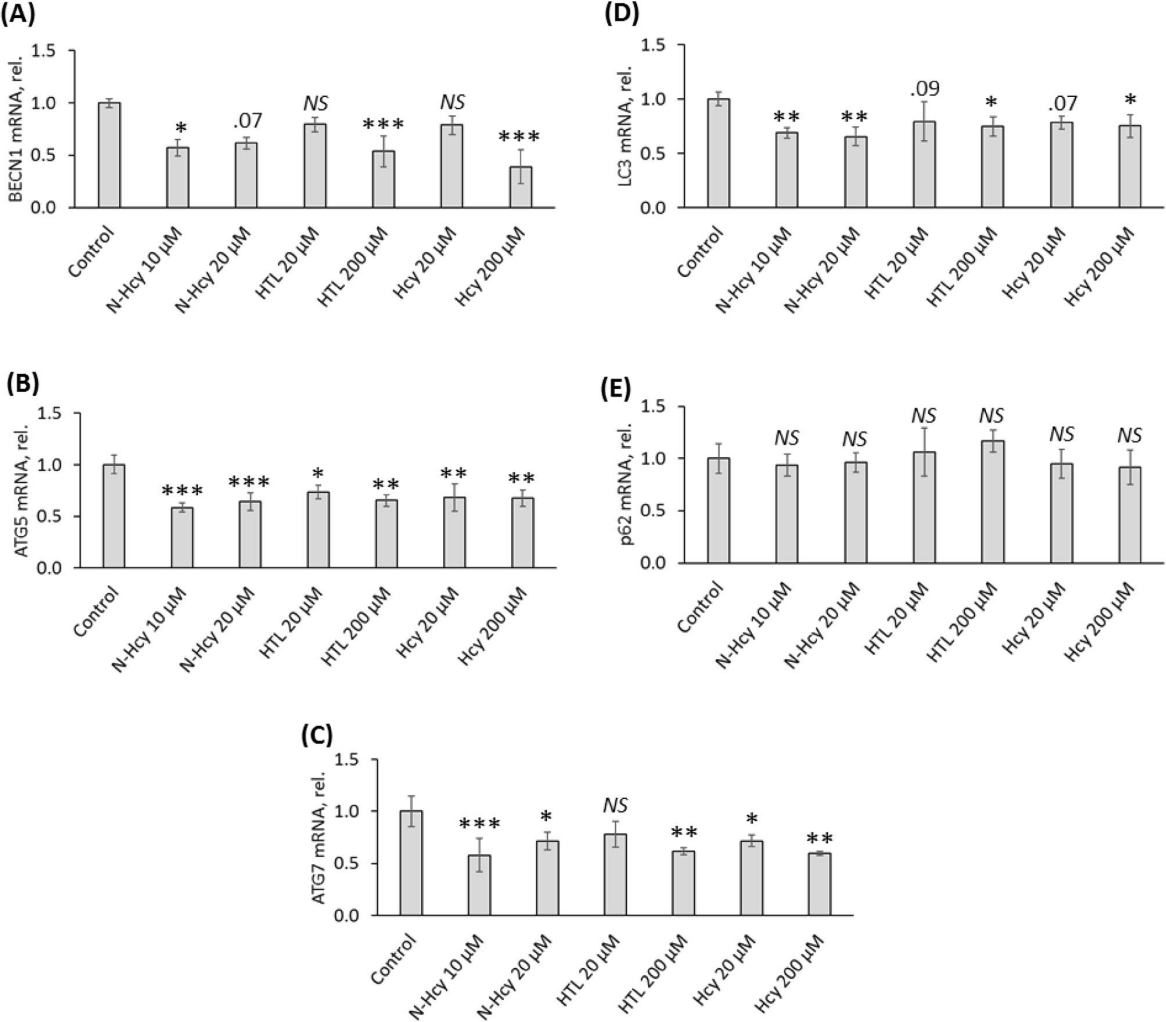
Influence of *N*-Hcy-protein, Hcy-thiolactone, and Hcy on the expression of autophagy-related mRNAs in HUVEC. Bar graphs show levels of BECN1 mRNA (**A**), ATG5 mRNA (**B**), ATG7 mRNA (**C**), LC3 mRNA (**D**), and p62 mRNA (**E**) in HUVEC treated with *N*-Hcy-protein (N-Hcy), Hcy-thiolactone (HTL), or Hcy in methionine-free M199/dialyzed FBS medium for 24 h. Showed mRNAs were quantified by RT-qPCR with GAPDH mRNA as a reference. Each assay was repeated three times (technical repeats) in three independent experiments (biological repeats). Mean ± SD values of three biological repeats for each treatment group are shown. *P < 0.05, **P < 0.01, ***P < 0.001 from one-way ANOVA with Tukey’s multiple comparisons test.

To elucidate whether effects of Hcy metabolites on the expression of autophagy-related proteins are transcriptional, we quantified by RT-qPCR mRNAs for these proteins in HUVECS treated with *N*-Hcy-protein, Hcy-thiolactone, and Hcy. We found significantly attenuated levels of BECN1 mRNA (Fig. 2A), ATG5 mRNA (Fig. 2B), and ATG7 mRNA (Fig. 2C) in HUVEC treated with *N*-Hcy-protein, Hcy-thiolactone, or Hcy, compared to untreated controls. Levels of LC3 mRNA were significantly downregulated by *N*-Hcy-protein, Hcy-thiolactone, and Hcy (Fig. 2D). p62 mRNA was unaffected by these metabolites (Fig. 2E). These findings show that Hcy metabolites exert transcriptional control over the expression of BECN1, ATG5, ATG7, and LC3 proteins.

### Hcy-thiolactone, *N*-Hcy-protein, and Hcy upregulate autophagy-related miR-21, mir-155, miR-216, and miR-320c

**Figure 3:**
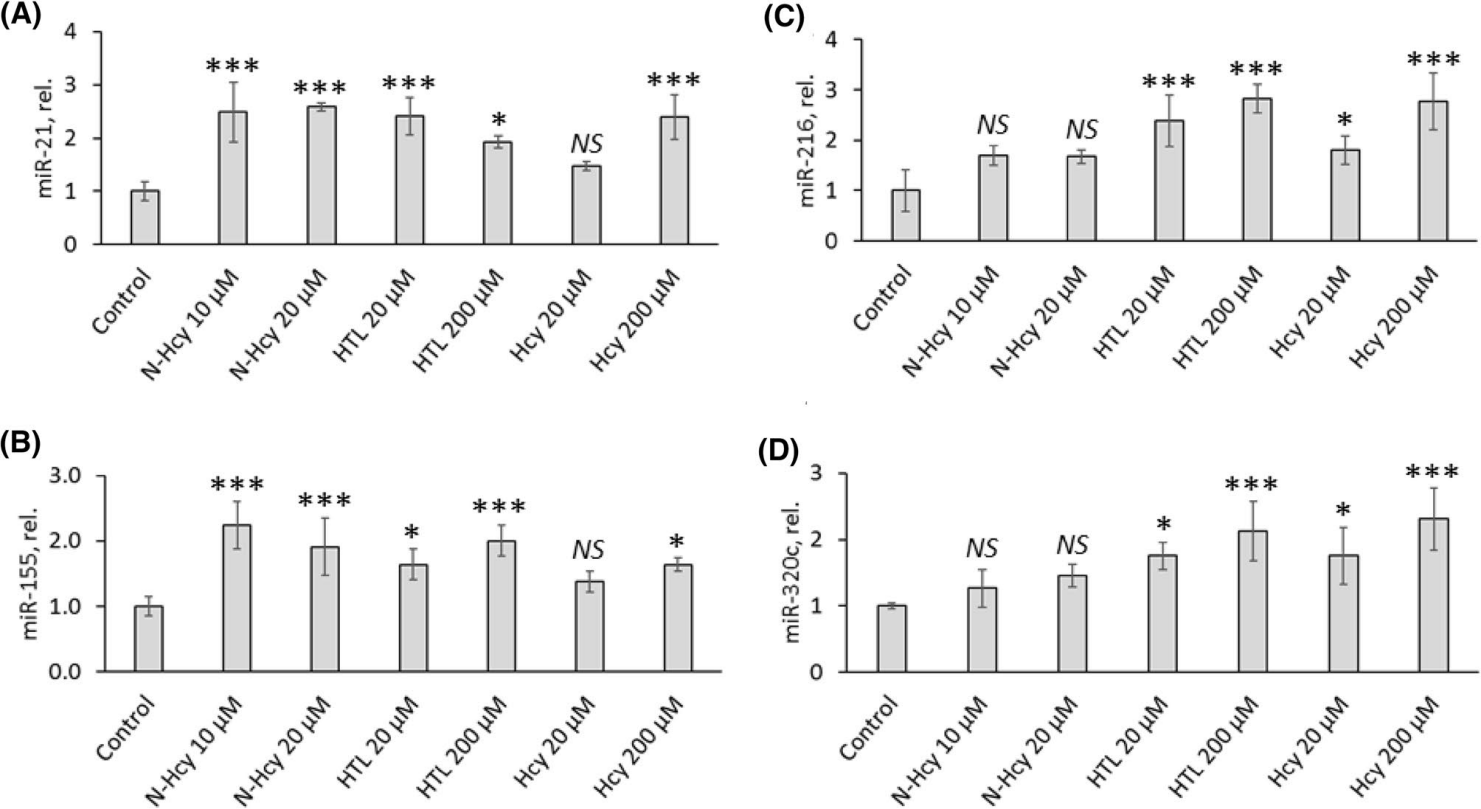
*N*-Hcy-protein, Hcy-thiolactone, and Hcy upregulate the expression of autophagy-related miRs in HUVEC. Bar graphs show levels of miR-21 (**A**), miR-155 (**B**), miR-216 (**C**), and miR-320c (**D**) in HUVEC treated with *N*-Hcy-protein (N-Hcy), Hcy-thiolactone (HTL), or Hcy for 24 h. The expression of miRs was quantified by RT-qPCR with 18S rRNA and U6 snRNA as references. Each assay was repeated three times (technical repeats) in three independent experiments (biological repeats). Mean ± SD values of three biological repeats for each treatment group are shown. *P < 0.05, **P < 0.01, ***P < 0.001 from one-way ANOVA with Tukey’s multiple comparisons test.

To find out whether the transcriptional downregulation of BECN1, ATG5, ATG7, and LC3 caused by Hcy metabolites is mediated by miRs targeting mRNAs encoding these proteins (https://mirtarbase.cuhk.edu.cn and Refs.^31–36^), we quantified miR-21, mir-155, miR-216, and miR-320c in HUVECs treated with Hcy-thiolactone, *N*-Hcy-protein, or Hcy. Target sites on BECN1, ATG5, and ATG7 mRNAs for these miRs have been validated by the dual-luciferase reporter vector analysis (miR-155-5p, Ref.^33^; miR-216b, Ref.^34^) or by miR-mRNA specific interaction using the crosslinking, ligation, and sequencing of hybrids (CLASH) method (miR-320c, Ref.^37^). We found significantly upregulated miR-21 (Fig. 3A), mir-155 (Fig. 3B), miR-216 (Fig. 3C), and miR-320c (Fig. 3D) levels in HUVECs treated with Hcy-thiolactone or Hcy compared to control. Treatments of HUVECs with *N*-Hcy-protein significantly elevated miR-21 (Fig. 3A) and mir-155 (Fig. 3B) levels. However, miR-216 (Fig. 3C) and miR-320c (Fig. 3D) were not affected by *N*-Hcy-protein.

### Treatments with miR inhibitors abrogate effects of Hcy-thiolactone, *N*-Hcy-protein, and Hcy on the levels of miR-21, mir-155, miR-216, and miR-320c

**Figure 4:**
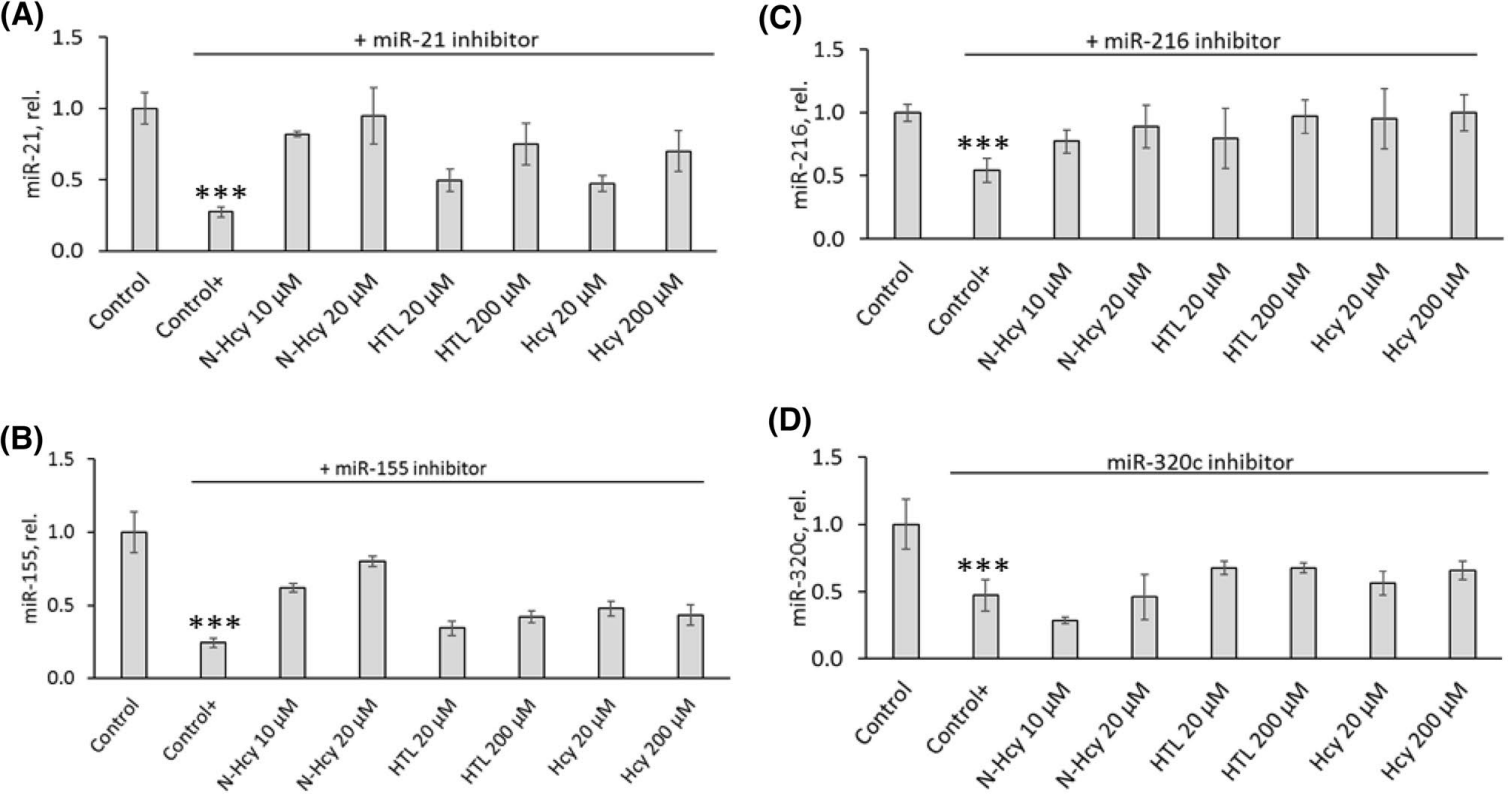
miR inhibitors abrogate stimulatory effects of *N*-Hcy-protein, Hcy-thiolactone, and Hcy on autophagy-related miR expression. Bar graphs show levels of miR-21 (**A**), miR-155 (**B**), miR-216 (**C**), and miR-320c (**D**) in HUVEC transfected with mirVana™ miRNA Mimic, Negative Control #1 (Control) or corresponding miR inhibitors for 4 h. The cells transfected with a miR inhibitor were then untreated (Control+) or treated with *N*-Hcy-protein (N-Hcy), Hcy-thiolactone (HTL), or Hcy in methionine-free M199/dialyzed FBS medium for 24 h. The expression of miRs was quantified by RT-qPCR with 18S rRNA and U6 snRNA as references. Each assay was repeated three times (technical repeats) in three independent experiments (biological repeats). Mean ± SD values of three biological repeats for each treatment group are shown. *P < 0.05, **P < 0.01, ***P < 0.001 from Student’s two-tailed *t* test.

To verify these findings, we conducted experiments with miR inhibitors. We found that transfections of HUVECs with miR-21 inhibitor significantly reduced miR-21 levels (to 27 ± 4% compared to untreated control, *P* < 0.0001; Fig. 4A). Transfections with miR-155 inhibitor reduced miR-155 levels (to 24 ± 3% compared to control, *P* < 0.0001; Fig. 4B). Transfections with miR-216 inhibitor reduced miR-216 levels (to 54 ± 10% compared to control, *P* < 0.0001; Fig. 4C). Transfections with miR-320c inhibitor reduced miR-320c levels (to 47 ± 12% compared to control, *P* < 0.0001; Fig. 4D).

At the same time, inhibitors of miR-21, miR-155, miR-216, or miR-320C abrogated the stimulatory effects of Hcy-thiolactone, *N*-Hcy-protein, and Hcy on miR-21 (Fig. 4A), mir-155 (Fig. 4B), miR-216 (Fig. 4C), and miR-320c (Fig. 4D) seen in the absence of these inhibitors (Fig. 3A–D, respectively).

**Figure 5:**
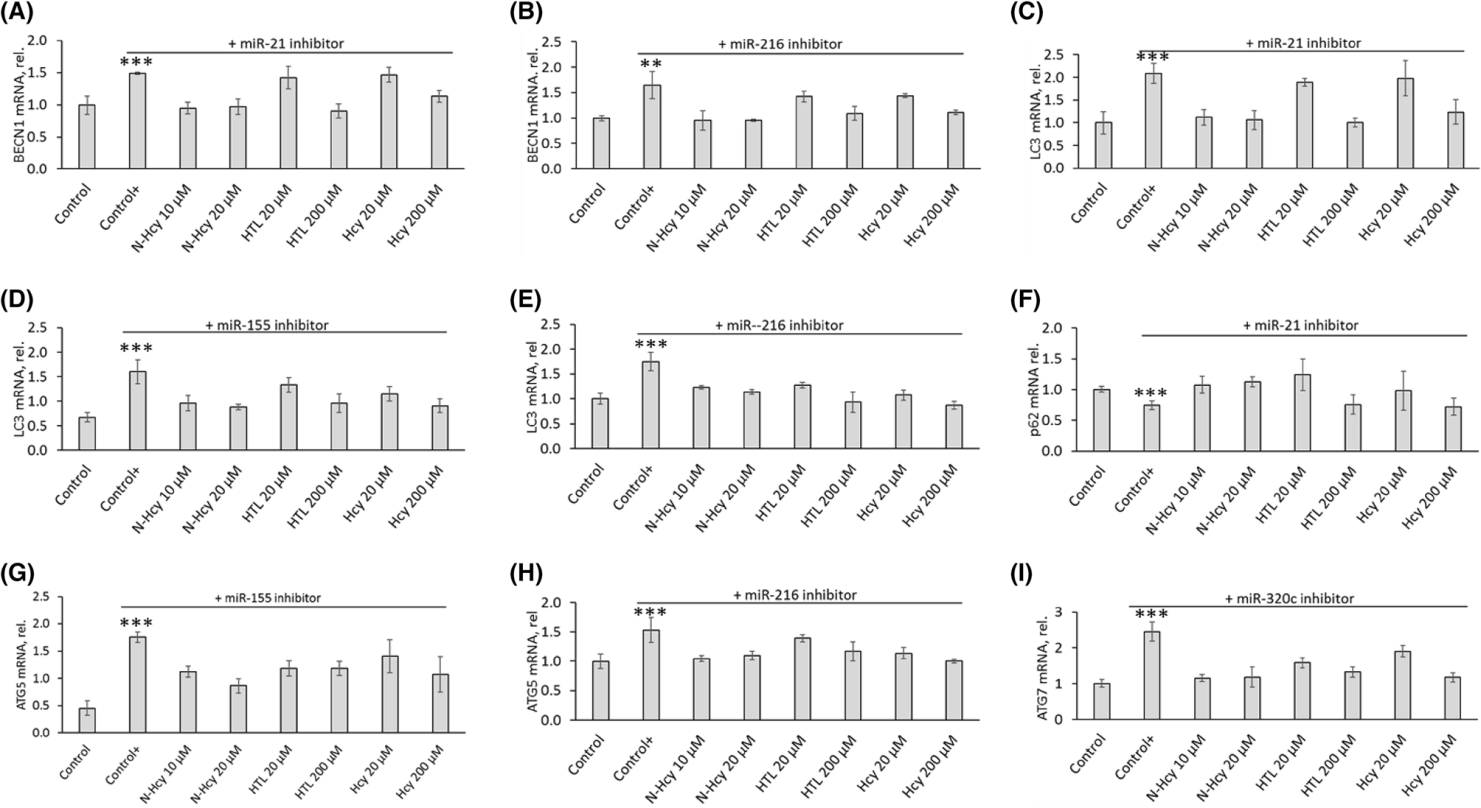
miR inhibitors abrogate stimulatory effects of *N*-Hcy-protein, Hcy-thiolactone, and Hcy on autophagy-related mRNA expression. Bar graphs show levels of BECN1 mRNA (**A**,**B**), LC3 mRNA (**C**–**E**), p62 mRNA (**F**), ATG5 mRNA (**G**,**H**), and ATG7 mRNA (**I**) in HUVEC treated with *N*-Hcy-protein (N-Hcy), Hcy-thiolactone (HTL), or Hcy in HUVEC transfected with mirVana™ miRNA Mimic, Negative Control #1 (Control) or corresponding miR inhibitors for 4 h. The cells transfected with a miR inhibitor were then untreated (Control+) or treated with *N*-Hcy-protein (N-Hcy), Hcy-thiolactone (HTL), or Hcy in methionine-free M199/dialyzed FBS medium for 24 h. Levels of mRNAs were quantified by RT-qPCR with GAPDH mRNA as a reference. Each assay was repeated three times (technical repeats) in three independent experiments (biological repeats). Mean ± SD values of three biological repeats for each treatment group are shown. *P < 0.05, **P < 0.01, ***P < 0.001 from Student’s two-tailed *t* test.

**Figure 6:**
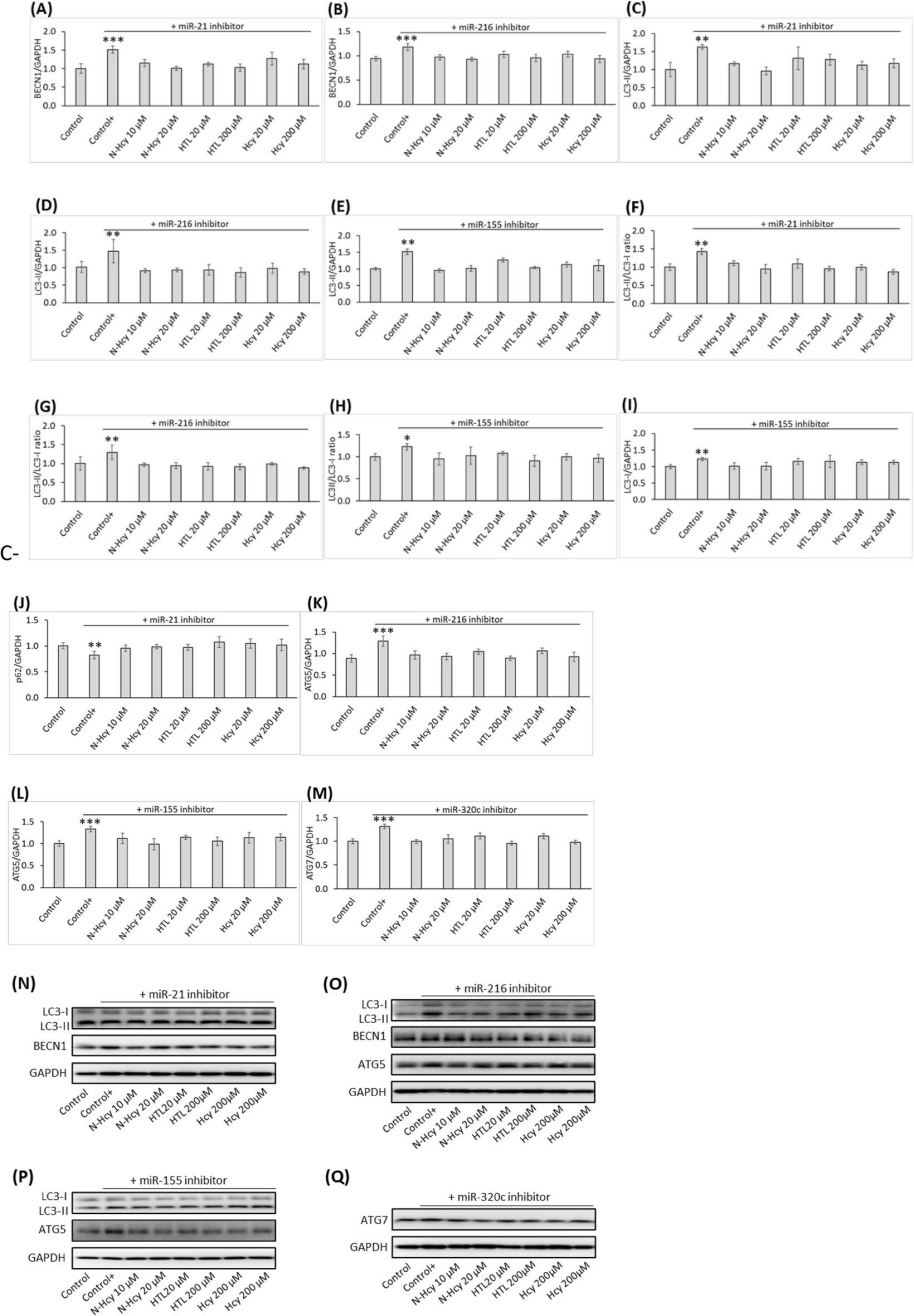
Influence of *N*-Hcy-protein, Hcy-thiolactone, and Hcy on the expression of autophagy-related proteins in HUVEC in the presence of a corresponding miR inhibitor. Bar graphs show levels of BECN1 protein (**A**,**B**), LC3-II protein (**C**–**E**), LC3-II/LC3-I ratio (**F**–**H**), LC3-I (**I**), p62 protein (**J**), ATG5 protein (**K**,**L**), and ATG7 protein (**M**) in HUVEC transfected with mirVana™ miRNA Mimic, Negative Control #1 (Control) or a corresponding miR inhibitor for 4 h. The cells transfected with a miR inhibitor were then untreated (Control+) or treated with N-Hcy, HTL, or Hcy in methionine-free M199/dialyzed FBS medium for 24 h. Indicated proteins were quantified by Western blotting with GAPDH protein as a reference; representative images are shown in panels (**N**–**Q**). Each assay was repeated three times (technical repeats) in three independent experiments (biological repeats). Mean ± SD values of three biological repeats for each treatment group are shown. *P < 0.05, **P < 0.01, ***P < 0.001 from a Student’s two-tailed *t* test.

We also found that treatments of HUVEC with miR-21 inhibitor or miR-216 inhibitor significantly increased BECN1 mRNA (*P* < 0.01; Fig. 5A,B) and BECN1 protein level (*P* < 0.001; Fig. 6A,B). Treatments with inhibitors of miR-21, miR-155, or miR-216 significantly increased LC3 mRNA (*P* < 0.001; Fig. 5C–E), LC3-II protein level (*P* < 0.01; Fig. 6C–E), and LC3-II/LC3-I ratio (*P* < 0.01; Fig. 6F–H), showing reduced autophagy flux. LC3-I protein level was significantly increased by miR-155 inhibitor (Fig. 6I) but was not affected by inhibitors of miR-21 and miR-216 (not shown).

Treatments with miR-21 inhibitor significantly decreased p62 mRNA (*P* < 0.001; Fig. 5F) and p62 protein level (*P* < 0.01; Fig. 6J). As p62 mRNA was not affected by treatments with Hcy metabolites (Fig. 2E) and because p62 and LC3 are inversely correlated^30^, this effect is most likely due to upregulation of LC3 by the miR-21 inhibitor.

Treatments with inhibitors of miR-155 or miR-216 significantly increased ATG5 mRNA (*P* < 0.001; Fig. 5G,H) and ATG5 protein level (*P* < 0.01; Fig. 6K,L) while treatments with mir-320c inhibitor significantly increased ATG7 mRNA (*P* < 0.001; Fig. 6I) and ATG7 protein level (*P* < 0.01; Fig. 6M). Representative western blot images for autophagy proteins quantification are shown in Fig. 6N–Q.

Inhibitors of miR-21, miR-155, miR-216, or miR-320C also abrogated the inhibitory effects of Hcy-thiolactone, *N*-Hcy-protein, and Hcy on BECN1, ATG5, ATG7, and LC3 mRNA and protein expression (Figs. 5 and 6, respectively) seen in the absence of miR inhibitors (mRNA, Fig. 2; protein, Fig. 1).

## Discussion

Endothelial dysfunction, the first step in the development of atherosclerosis, plays a central role in CVD and is a common finding in HHcy in humans and in animal models^3,4^. To understand the mechanisms by which HHcy disrupts normal cellular function and causes disease, we studied in HUVECs, a widely used model of vascular cells^10,37^, how Hcy, Hcy-thiolactone and *N*-Hcy-protein, metabolites that accumulate in HHcy, affect the expression of miR-21, miR-155, miR-216, and miR-320c, regulators of the expression of BECN1, ATG5, ATG7, LC3, and p62 genes (Fig. 3) participating in autophagy, a process important for vascular homeostasis^37^. Our data show that Hcy, Hcy-thiolactone, and *N*-Hcy-protein dysregulate autophagy by upregulating the expression of miR-21, miR-155, miR-216, and miR-320c in HUVECs. The ability of Hcy, Hcy-thiolactone, and *N*-Hcy-protein to upregulate miR-21, miR-155, miR-216, and miR-320c expression and thus affect autophagy can explain the susceptibility of human endothelial cells to HHcy-induced endothelial dysfunction and atherosclerosis.

Studies of Hcy metabolism led to the discoveries of Hcy-thiolactone and *N*-Hcy-protein in human cells and the proposal by one of us (HJ) that these metabolites are responsible for the pathologies associated with HHcy^38^. The metabolic conversions of Hcy to Hcy-thiolactone and *N*-Hcy-protein are universal and were confirmed in HUVEC^10^, mice^39,40^, and humans^39,41,42^. More recently, clinical studies by one of us (HJ) have shown that HTL predicts a risk of myocardial infarction in patients with coronary artery disease, thereby lending a support to the hypothesis that Hcy-thiolactone is mechanistically involved in CVD^43^. Our present findings that Hcy-thiolactone, *N*-Hcy-protein, and Hcy, upregulate miR-21, miR-155, miR-216, and miR-320c in HUVECs and inhibit autophagy, suggest that these miRs can also be involved in endothelial dysfunction and suggest a mechanism underlying the involvement of HHcy in CVD.

Our present findings that the expression of BECN1, ATG5, ATG7, and LC3 as well as the LC3-II/LC3-I ratio, the measure of autophagy flux, are downregulated by Hcy, Hcy-thiolactone, and *N*-Hcy-protein in HUVEC suggest that impaired autophagy can accelerate endothelial dysfunction and lead to vascular disease. Indeed, other investigators have shown that autophagy flux controls endothelial cells homeostasis while impaired autophagy due to factors other than HHcy can promote pro-atherogenic phenotype^37^.

Over the past two decades, miRs have been identified as key regulators of the pathophysiological processes involved in atherosclerosis such as signaling (NF-κB, MAPK, SOCS, SDF-1/CXCR4, TGF-β, BMP, TLR) and lipid homeostasis pathways^44^. The HDL-carried miRNAs can mediate extracellular signaling by repressing genes in target tissues and HDL interaction with macrophages and endothelial cells may also result in miR exchange. Our present findings show that, Hcy metabolites, which are associated with CVD, upregulate miRs that target autophagy in HUVEC: mir-21 (affecting *LC3 BECN1*, and *ATG3* mRNA) in bovine granulosa cells^31^; mir-155 targeting ATG5 that also affects LC3 and p62 expression^32–34^ in HUVEC; miR-216 targeting *BECN1* and *ATG5* mRNAs^35^ https://mirtarbase.cuhk.edu.cn/~miRTarBase/miRTarBase_2022/php/detail.php?mirtid=MIRT054887; and miR-320c targeting ATG7^36^ https://mirtarbase.cuhk.edu.cn/~miRTarBase/miRTarBase_2022/php/detail.php?mirtid=MIRT036161.

Among these, miR-216a (targeting *BECN1*), highly expressed in arteries, is induced during endothelial aging and controls oxidized low density lipoprotein (ox-LDL)-induced autophagy in HUVEC by regulating levels of BECN1^35^; miR-155 (targeting ATG5) that also affects LC3 and p62 expression, promotes ox-LDL-induced^34^ and hydrogen peroxide-induced^33^ autophagy in HUVEC; miR-320c (targeting ATG7) is associated with aging and cancer^36^. Our present findings showing that *N*-Hcy-protein, Hcy-thiolactone, and Hcy upregulate miR-21, miR-155, miR-216, and miR-320c suggest that miR-dependent dysregulation of autophagy can be involved in endothelial dysfunction and atherosclerosis induced by HHcy.

The concentrations of Hcy and related metabolites used in the present study did not affect HUVEC viability (Fig. 1I). However, these concentrations of Hcy are known to induce the endothelial adhesion molecule VCAM-1 expression, characteristic of endothelial activation/dysfunction, without affecting cell viability^6^; much higher Hcy concentrations can cause cell death^45^.

In conclusion, our findings define a new miR-mediated mechanism by which HHcy can induce endothelial dysfunction. In this mechanism, Hcy, Hcy-thiolactone, and *N*-Hcy-protein, metabolites associated with HHcy, upregulate the expression of miR-21, miR-155, miR-216, and miR-320c, which results in impaired autophagy.

## Materials and methods

### Cell culture and treatments

Human primary umbilical vein endothelial cells (HUVEC; ATCC, Manassas, VA, USA, Cat. # PCS-100-013) were seeded in 25-cm^2^ flasks in Vascular Cell Basal Medium (ATCC, Cat. # PCS-100-030) supplemented with 5% FBS, Endothelial Cell Growth Kit-VEGF (ATCC), antibiotics (penicillin/streptomycin) (MilliporeSigma, Saint Louis, MO, USA), and grown in 5% CO_2_ atmosphere at 37 °C.

For the Hcy metabolite treatment experiments, HUVEC monolayers were trypsinized, washed with PBS, seeded in 6-well plates (100,000 cells/well), and grown as above. After cells reached 70–80% confluency, the monolayers were washed with PBS (2-times) and overlaid with M199 medium without methionine (Thermo Fisher Scientific, Waltham, MA, USA) and with 5% dialyzed fetal bovine serum (FBS; Millipore Sigma). Cell cultures were treated with *N*-Hcy-protein (prepared as described in Ref.^11^), L-Hcy-thiolactone, or D,L-Hcy (Millipore Sigma) (at concentrations based on earlier work^10,11^ and indicated in the figures) and incubated at 37 °C in 5% CO_2_ for 24 h; cells from untreated cultures were used as controls. These concentrations reflect levels of these metabolites in mice and humans (reviewed in Ref.^7^).

For the miR inhibition studies, HUVEC were transfected in Opti-MEM medium (Thermo Scientific) with 0.5 nM specific miR inhibitors (MH10203 for hsa-miR-22-3p or MH13382 for hsa-miR-1229-3p; Thermo Scientific) or 0.5 nM mirVana™ miR Mimic, Negative Control #1 (Thermo Scientific) as a control using Lipofectamine RNAiMax. After a 4-h-incubation, cell monolayers were washed with PBS (2-times), overlaid with M199 medium without methionine (Thermo Scientific) containing 5% dialyzed FBS (Millipore Sigma), and treated with D, L-Hcy, L-Hcy-thiolactone (Millipore Sigma), or *N*-Hcy-protein as above.

### HUVEC viability assay

The viability of HUVEC was assessed by using the trypan blue exclusion assay based on the principle that the dye can enter membrane-compromised dead cells but is excluded by live cells. Briefly, 10,000 cells/well were seeded into wells of a 48-well plate. After cells reached 70–80% confluency (at 24–48 h), cells were treated as described in “Hcy-thiolactone, N-Hcy-protein, and Hcy downregulate autophagy-related proteins” section Cell culture and treatments. After 24 h, cells were rinsed with PBS (2-times), trypsinized, and pelleted by centrifuged (5 min, RT, 1500 rpm). Cell pellets were suspended and incubated for 3 min in 350 µl PBS mixed with 150 µl 0.4% trypan blue solution (Millipore Sigma). For quantifying viability, cells were transferred to a hemocytometer chamber and counted using a light micrroscope at 10 × magnification. The HUVEC viability is expressed as the percentage of blue-stained dead cells in the total number of cells.

### Western blots

Whole cell lysates of 300,000 to 40,000 cells/well in RIPA buffer (Millipore Sigma) were prepared according to manufacturer’s protocol. Protein concentration was measured using Pierce™ BCA protein Kit (Termo Fisher). Following separation on SDS-PAGE 10% gels (8 µg protein/lane, 5% total), proteins transferred to PVDF membrane (Bio-Rad) for 20 min at 0.1 A, 25 V using Trans Blot Turbo Transfer System (Bio-Rad) as described earlier^29,46^. After blocking with 5% bovine serum albumin in TBST buffer (1 h, room temperature), the membranes were incubated with relevant primary antibodies: anti-ATG5 (CS, #12994), anti-ATG7 (CS, #8558), anti-BECN1 (CS, #3495), anti-p62 (CS, #39749), anti-LC3 (CS, #12741), and anti-Gapdh (CS, #5174), followed by horseradish peroxidase-conjugated goat anti-rabbit IgG secondary antibody (Cell Signaling Technology) for 16 h. Images were captured using Western Bright Quantum-Advansta K12042-D20 and GeneGnome XRQ NPC chemiluminescence detection system. Band intensities were calculated using the Gene Tools program from Syngene.

### Quantification of mRNA and miR by RT-qPCR

Total RNA was isolated using Trizol reagent (Millipore Sigma). cDNA synthesis was performed using Revert Aid First cDNA Synthesis Kit (Thermo Fisher Scientific) according to manufacturer’s instructions. RNA concentration was measured using NanoDrop (Thermo Fisher Scientific). RT-qPCR was performed using SYBR Green Mix and CFX96 thermocycler (Bio-Rad) and primers listed in Supplementary Table S1.

The miR 1st-Strand cDNA Synthesis Kit (Agilent Technologies) was used according to manufacturer’s instructions to polyadenylate and reverse-transcribe miRs from one μg of total RNA. To quantify miR levels, RT-qPCR was performed with the resulting cDNA in miRNA QPCR Master Mix (Agilent Technologies) using a universal reverse primer (Agilent Technologies) and unique miR-specific primers (same sequence as an analyzed miR) (Supplementary Table S1). Reactions were conducted on CFX96 thermocycler (Bio-Rad). 18S rRNA and U6 snRNA were used as references for miR quantification.

The 2^(−ΔΔCt)^ method was used to calculate the relative expression levels^47^. Data analysis was performed with the CFX Manager™ Software, Microsoft Excel, and GraphPad Prism7.

### Statistical analysis

Each assay was repeated three times (technical repeats) in three independent experiments (biological repeats) for each treatment and controls. Data are mean ± standard deviation (SD) of three biological repeats. Data were analyzed using one-way analysis of variance (ANOVA) with Tukey’s multiple comparisons post-test or a Student t test using GraphPad Prism7 software (GraphPad Holdings LLC, San Diego CA, USA, https://www.graphpad.com).

### Supplementary Information

Supplementary Information 1.

Supplementary Information 2.

## Data Availability

All data generated or analyzed during this study are included in this published article. Correspondence and requests for materials should be addressed to H.J.

## References

[1] Libby P (2002). Inflammation in atherosclerosis. Nature.

[2] Ross R (1999). Atherosclerosis—An inflammatory disease. N. Engl. J. Med..

[3] Lentz SR (2005). Mechanisms of homocysteine-induced atherothrombosis. J. Thromb. Haemost..

[4] Dayal S, Lentz SR (2008). Murine models of hyperhomocysteinemia and their vascular phenotypes. Arterioscler. Thromb. Vasc. Biol..

[5] Esse R, Barroso M, Tavares de Almeida I, Castro R (2019). The contribution of homocysteine metabolism disruption to endothelial dysfunction: State-of-the-art. Int. J. Mol. Sci..

[6] Carluccio MA, Ancora MA, Massaro M, Carluccio M, Scoditti E, Distante A, Storelli C, De Caterina R (2007). Homocysteine induces VCAM-1 gene expression through NF-kappaB and NAD(P)H oxidase activation: Protective role of Mediterranean diet polyphenolic antioxidants. Am. J. Physiol. Heart Circ. Physiol..

[7] Jakubowski H (2019). Homocysteine modification in protein structure/function and human disease. Physiol. Rev..

[8] Sikora M, Lewandowska I, Marczak L, Bretes E, Jakubowski H (2020). Cystathionine beta-synthase deficiency: Different changes in proteomes of thrombosis-resistant Cbs^(^^−^^/^^−^^)^ mice and thrombosis-prone CBS^(^^−^^/^^−^^)^ humans. Sci. Rep..

[9] Sikora M, Jakubowski H (2021). Changes in redox plasma proteome of Pon1^−^^/^^−^ mice are exacerbated by a hyperhomocysteinemic diet. Free Radic. Biol. Med..

[10] Jakubowski H, Zhang L, Bardeguez A, Aviv A (2000). Homocysteine thiolactone and protein homocysteinylation in human endothelial cells: Implications for atherosclerosis. Circ. Res..

[11] Gurda D, Handschuh L, Kotkowiak W, Jakubowski H (2015). Homocysteine thiolactone and N-homocysteinylated protein induce pro-atherogenic changes in gene expression in human vascular endothelial cells. Amino Acids.

[12] Olejniczak M, Urbanek MO, Jaworska E, Witucki L, Szczesniak MW, Makalowska I, Krzyzosiak WJ (2016). Sequence-non-specific effects generated by various types of RNA interference triggers. Biochim. Biophys. Acta.

[13] Starega-Roslan J, Krzyzosiak WJ (2013). Analysis of microRNA length variety generated by recombinant human Dicer. Methods Mol. Biol..

[14] Starega-Roslan J, Galka-Marciniak P, Krzyzosiak WJ (2015). Nucleotide sequence of miRNA precursor contributes to cleavage site selection by Dicer. Nucleic Acids Res..

[15] Starega-Roslan J, Koscianska E, Kozlowski P, Krzyzosiak WJ (2011). The role of the precursor structure in the biogenesis of microRNA. Cell. Mol. Life Sci..

[16] Stroynowska-Czerwinska A, Fiszer A, Krzyzosiak WJ (2014). The panorama of miRNA-mediated mechanisms in mammalian cells. Cell. Mol. Life Sci..

[17] Minjares M, Wu W, Wang JM (2023). Oxidative stress and microRNAs in endothelial cells under metabolic disorders. Cells.

[18] Zhou SS, Jin JP, Wang JQ, Zhang ZG, Freedman JH, Zheng Y, Cai L (2018). miRNAS in cardiovascular diseases: Potential biomarkers, therapeutic targets and challenges. Acta Pharmacol. Sin..

[19] Mens MMJ, Heshmatollah A, Fani L, Ikram MA, Ikram MK, Ghanbari M (2021). Circulatory microRNAs as potential biomarkers for stroke risk: The Rotterdam study. Stroke.

[20] Sobering AK, Bryant LM, Li D, McGaughran J, Maystadt I, Moortgat S, Graham JM, van Haeringen A, Ruivenkamp C, Cuperus R, Vogt J, Morton J, Brasch-Andersen C, Steenhof M, Hansen LK, Adler E, Lyonnet S, Pingault V, Sandrine M, Ziegler A, Donald T, Nelson B, Holt B, Petryna O, Firth H, McWalter K, Zyskind J, Telegrafi A, Juusola J, Person R, Bamshad MJ, Earl D, Tsai AC, Yearwood KR, Marco E, Nowak C, Douglas J, Hakonarson H, Bhoj EJ, University of Washington Center for Mendelian Genomics (2022). Variants in PHF8 cause a spectrum of X-linked neurodevelopmental disorders and facial dysmorphology. HGG Adv.

[21] Laumonnier F, Holbert S, Ronce N, Faravelli F, Lenzner S, Schwartz CE, Lespinasse J, Van Esch H, Lacombe D, Goizet C, Phan-Dinh Tuy F, van Bokhoven H, Fryns JP, Chelly J, Ropers HH, Moraine C, Hamel BC, Briault S (2005). Mutations in PHF8 are associated with X linked mental retardation and cleft lip/cleft palate. J. Med. Genet..

[22] Vargas JNS, Hamasaki M, Kawabata T, Youle RJ, Yoshimori T (2023). The mechanisms and roles of selective autophagy in mammals. Nat. Rev. Mol. Cell. Biol..

[23] Kim KA, Shin D, Kim JH, Shin YJ, Rajanikant GK, Majid A, Baek SH, Bae ON (2018). Role of autophagy in endothelial damage and blood–brain barrier disruption in ischemic stroke. Stroke.

[24] Lin X, Ouyang S, Zhi C, Li P, Tan X, Ma W, Yu J, Peng T, Chen X, Li L, Xie W (2022). Focus on ferroptosis, pyroptosis, apoptosis and autophagy of vascular endothelial cells to the strategic targets for the treatment of atherosclerosis. Arch Biochem. Biophys..

[25] Jeong SJ, Oh GT (2023). Unbalanced redox with autophagy in cardiovascular disease. J. Lipid Atheroscler..

[26] Khayati K, Antikainen H, Bonder EM, Weber GF, Kruger WD, Jakubowski H, Dobrowolski R (2017). The amino acid metabolite homocysteine activates mTORC1 to inhibit autophagy and form abnormal proteins in human neurons and mice. FASEB J..

[27] Witucki L, Jakubowski H (2023). Homocysteine metabolites inhibit autophagy, elevate amyloid beta, and induce neuropathy by impairing Phf8/H4K20me1-dependent epigenetic regulation of mTOR in cystathionine β-synthase-deficient mice.. J Inherit Metab Dis.

[28] Witucki L, Borowczyk K, Suszyńska-Zajczyk J, Warzych E, Pawlak P, Jakubowski H (2023). Deletion of the homocysteine thiolactone-detoxifying enzyme bleomycin hydrolase, in mice, causes memory and neurological deficits and worsens Alzheimer's disease-related behavioral and biochemical traits in the 5xFAD model of Alzheimer's disease.. J Alzheimer's Dis.

[29] Witucki L, Jakubowski H (2023). Depletion of paraoxonase 1 (Pon1) dysregulates mTOR, autophagy, and accelerates amyloid beta accumulation in mice. Cells.

[30] Min Z, Ting Y, Mingtao G, Xiaofei T, Dong Y, Chenguang Z, Wei D (2018). Monitoring autophagic flux using p62/SQSTM1 based luciferase reporters in glioma cells. Exp. Cell Res..

[31] Ma L, Zheng Y, Tang X, Gao H, Liu N, Gao Y, Hao L, Liu S, Jiang Z (2019). miR-21-3p inhibits autophagy of bovine granulosa cells by targeting VEGFA via PI3K/AKT signaling. Reproduction.

[32] Yu Q, Xu XP, Yin XM, Peng XQ (2021). miR-155-5p increases the sensitivity of liver cancer cells to adriamycin by regulating ATG5-mediated autophagy. Neoplasma.

[33] Chen H, Liu Gao MY, Zhang L, He FL, Shi YK, Pan XH, Wang H (2019). MicroRNA-155 affects oxidative damage through regulating autophagy in endothelial cells. Oncol. Lett..

[34] Yin S, Yang S, Pan X, Ma A, Ma J, Pei H, Dong Y, Li S, Li W, Bi X (2018). MicroRNA-155 promotes ox-LDL-induced autophagy in human umbilical vein endothelial cells by targeting the PI3K/Akt/mTOR pathway. Mol. Med. Rep..

[35] Menghini R, Casagrande V, Marino A, Marchetti V, Cardellini M, Stoehr R, Rizza S, Martelli E, Greco S, Mauriello A, Ippoliti A, Martelli F, Lauro R, Federici M (2014). MiR-216a: A link between endothelial dysfunction and autophagy. Cell Death Dis..

[36] Helwak A, Kudla G, Dudnakova T, Tollervey D (2013). Mapping the human miRNA interactome by CLASH reveals frequent noncanonical binding. Cell.

[37] Mameli E, Martello A, Caporali A (2022). Autophagy at the interface of endothelial cell homeostasis and vascular disease. FEBS J..

[38] Jakubowski H (1997). Metabolism of homocysteine thiolactone in human cell cultures. Possible mechanism for pathological consequences of elevated homocysteine levels. J. Biol. Chem..

[39] Chwatko G, Boers GH, Strauss KA, Shih DM, Jakubowski H (2007). Mutations in methylenetetrahydrofolate reductase or cystathionine beta-synthase gene, or a high-methionine diet, increase homocysteine thiolactone levels in humans and mice. Faseb J..

[40] Jakubowski H, Perla-Kajan J, Finnell RH, Cabrera RM, Wang H, Gupta S, Kruger WD, Kraus JP, Shih DM (2009). Genetic or nutritional disorders in homocysteine or folate metabolism increase protein N-homocysteinylation in mice. Faseb J..

[41] Jakubowski H, Boers GH, Strauss KA (2008). Mutations in cystathionine {beta}-synthase or methylenetetrahydrofolate reductase gene increase N-homocysteinylated protein levels in humans. FASEB J..

[42] Perla-Kajan J, Stanger O, Luczak M, Ziolkowska A, Malendowicz LK, Twardowski T, Lhotak S, Austin RC, Jakubowski H (2008). Immunohistochemical detection of N-homocysteinylated proteins in humans and mice. Biomed. Pharmacother..

[43] Borowczyk K, Piechocka J, Glowacki R, Dhar I, Midtun O, Tell GS, Ueland PM, Nygard O, Jakubowski H (2019). Urinary excretion of homocysteine thiolactone and the risk of acute myocardial infarction in coronary artery disease patients: The WENBIT trial. J. Intern. Med..

[44] Feinberg MW, Moore KJ (2016). MicroRNA regulation of atherosclerosis. Circ. Res..

[45] Zhang C, Cai Y, Adachi MT, Oshiro S, Aso T, Kaufman RJ, Kitajima S (2001). Homocysteine induces programmed cell death in human vascular endothelial cells through activation of the unfolded protein response. J. Biol. Chem..

[46] Witucki L, Kurpik M, Jakubowski H, Szulc M, Lukasz Mikolajczak P, Jodynis-Liebert J, Kujawska M (2022). Neuroprotective effects of cranberry juice treatment in a rat model of Parkinson's disease. Nutrients.

[47] Livak KJ, Schmittgen TD (2001). Analysis of relative gene expression data using real-time quantitative PCR and the 2(−Delta Delta C(T)) method. Methods.

