# Supplementary material for: Homocysteine metabolites inhibit autophagy by upregulating miR-21-5p, miR-155-5p, miR-216-5p, and miR-320c-3p in human vascular endothelial cells
Source: Sci Rep. 2024 Mar 26;14:7151. doi: 10.1038/s41598-024-57750-3 (PMC10966103; doi:10.1038/s41598-024-57750-3)
Supplement: Supplementary file 1 — Supplementary Information 1. [file 41598_2024_57750_MOESM1_ESM.docx]

**Homocysteine Metabolites Inhibit Autophagy by Upregulating miR-21-5p, miR-155-5p, miR-216-5p, and miR-320c-3p in Human Vascular Endothelial Cells**

**Łukasz Witucki ^1^ and Hieronim Jakubowski  ^1, 2,^ ***

**^1^** Department of Biochemistry and Biotechnology, Poznań University of Life Sciences, 60-632 Poznań, Poland;

^2^ Department of Microbiology, Biochemistry and Molecular Genetics, Rutgers University, New Jersey Medical School, International Center for Public Health, Newark, NJ 07103, USA

***** Correspondence: jakubows@rutgers.edu, Tel: 973‐972‐8733; Fax: 973‐972‐8981.

**Supplementary Material**

**Supplementary Figure S1**

**Supplementary Table S1**

**
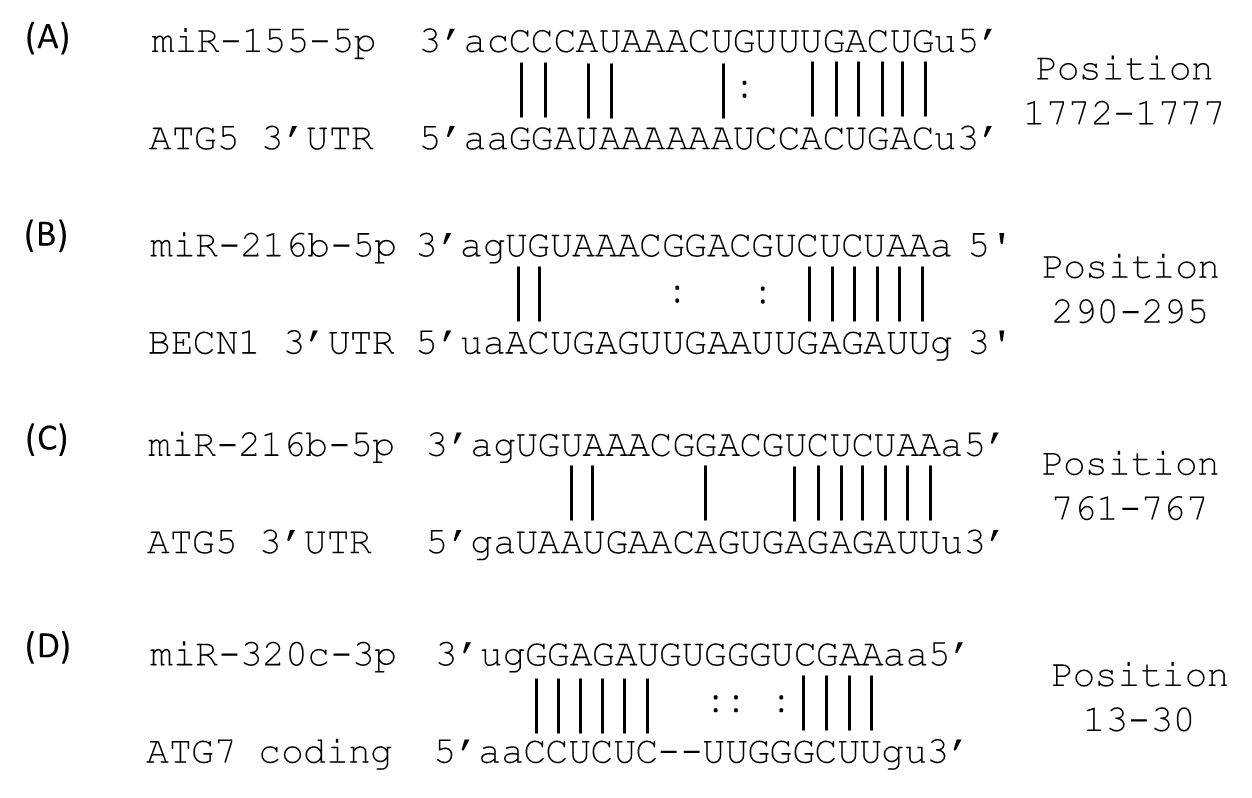
**

**Supplementary Figure S1.** Validated 3’UTR or coding sequence target sites for miRs in autophagy-related mRNAs. The ATG5 site for miR-155-5p (position 1772–1797) and BECN1 site for miR-216-5p (position 290-295) have been validated by the dual-luciferase reporter vector analysis (ref. 33, 34). The ATG5 site for miR-216b-5p (position 761-767) and ATG7 site for miR-320c-5p (position 13-30) have been validated by miR-mRNA specific interaction using the crosslinking, ligation, and sequencing of hybrids (CLASH) method (ref. 37) (<https://mirtarbase.cuhk.edu.cn/~miRTarBase/miRTarBase_2022/php/detail.php?mirtid=MIRT036161> ) (<https://www.targetscan.org/cgi-bin/targetscan/vert_80/view_gene.cgi?rs=ENST00000360666.4&taxid=9606&members=miR-216b-5p&showcnc=0&shownc=0&subset=1>).

| **Supplementary *Table S1.*** *Primers used for RT-qPCR* | |
| --- | --- |
| *Gene* | *Primer sequence* |
| *BECN1* | *Forward: 5′-GGGCTCCCGAGGGATGG-3′* |
|  | *Reverse: 5′-TTCCTCCTGGGTCTCTCCTG-3′* |
| *ATG5* | *Forward: 5′-AGTAGTTGCCTGGAGGAGCG-3′* |
|  | *Reverse: 5′-GTCTCGCCAACTCCACCTTG-3′* |
| *ATG7* | *Forward: 5′-GATGAAGCTCCCAAGGACAT-3′* |
|  | *Reverse: 5′-GTGGGAGCACTCATGTCAAA-3′* |
| *LC3* | *Forward: 5′-CATGAGCGAGTTGGTCAAGA-3′* |
|  | *Reverse: 5′-CCATGCTGTGCTGGTTCA-3′* |
| *P62* | *Forward: 5′-GGTCGCGCTCACCTTTCT-3′* |
|  | *Reverse: 5′-TCCTTTCTCAAGCCCCATGTT-3′* |
| *GAPDH* | *Forward: 5′-AGCCACATCGCTCAGACAC-3′* |
|  | *Reverse: 5′-GCCAATACGACCAAATCC-3′* |
| *miR-21-5p* | *5′-TAGCTTATCAGACTGATGTTGA-3′* |
| *miR-155-5p* | *5′-TTAATGCTAATCGTGATAGGGGTT-3′* |
| *miR-216b-5p* | *5′-AAATCTCTGCAGGCAAATGTGA-3′* |
| *miR-320c-3p* | *5′-AAAAGCTGGGTTGAGAGGGT-3′* |
| *18s rRNA* | *Reverse: 5′-AGGAATTCCCAGTAAGTGCG-3′* |
|  | *Reverse: 5′-GCCTCACTAAACCATCCAA-3′* |
| *U6 snRNA* | *Reverse: 5′-CTCGCTTCGGCAGCACA-3′* |
|  | *Reverse: 5′-AACGCTTCACGAATTTGCGT-3′* |
