# Supplementary material for: Homocysteine metabolites inhibit autophagy by upregulating miR-21-5p, miR-155-5p, miR-216-5p, and miR-320c-3p in human vascular endothelial cells
Source: Sci Rep. 2024 Mar 26;14:7151. doi: 10.1038/s41598-024-57750-3 (PMC10966103; doi:10.1038/s41598-024-57750-3)
Supplement: Supplementary file 2 — Supplementary Information 2. [file 41598_2024_57750_MOESM2_ESM.pptx]

## Slide 1
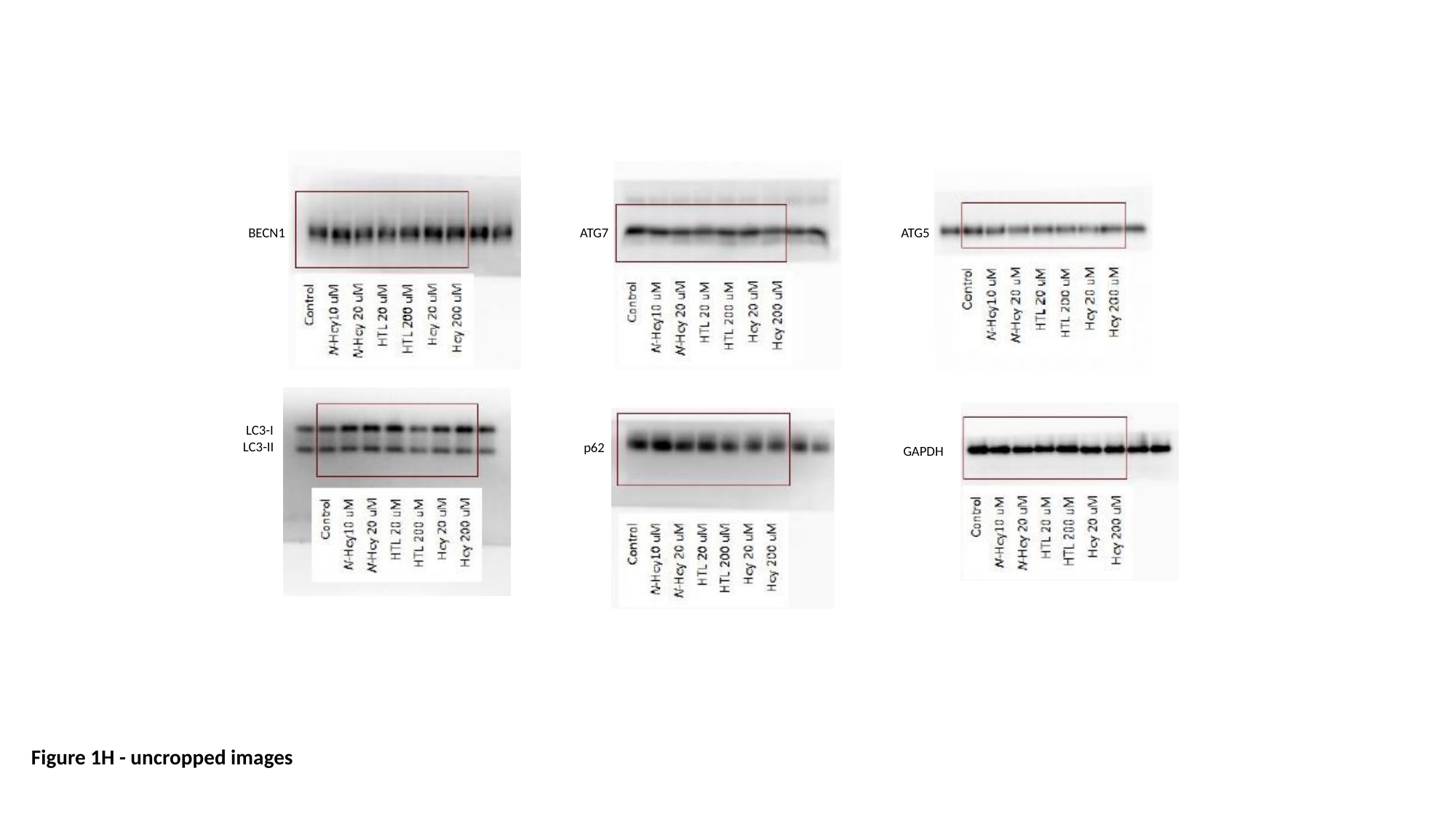

BECN1
ATG7
ATG5
 LC3-I
LC3-II
p62
GAPDH
Figure 1H - uncropped images

## Slide 2
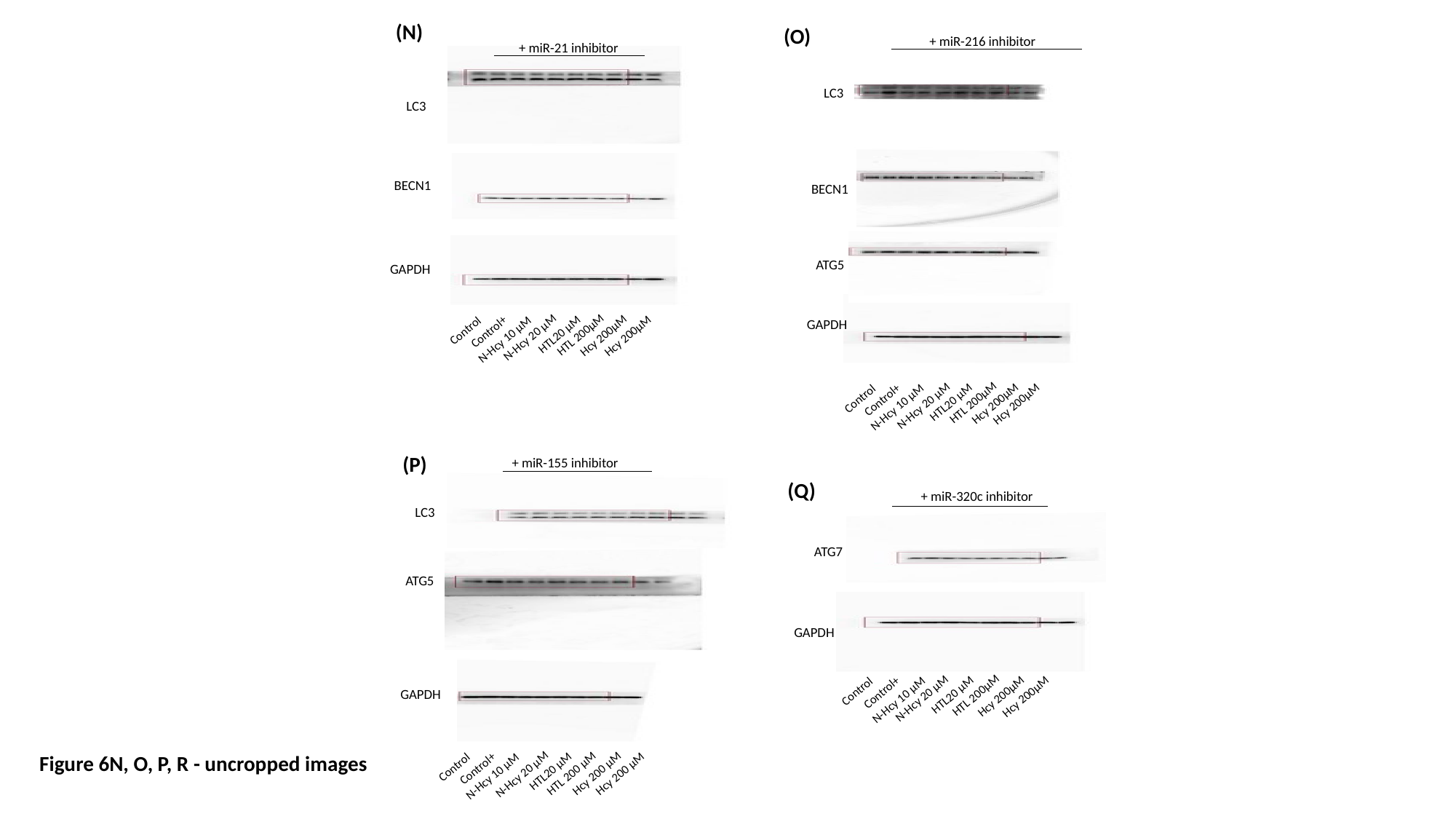

(N)
(O)
+ miR-216 inhibitor
+ miR-21 inhibitor
LC3
LC3
BECN1
BECN1
ATG5
GAPDH
GAPDH
Control
Control+
HTL20 µM
HTL 200µM
Hcy 200µM
Hcy 200µM
N-Hcy 20 µM
N-Hcy 10 µM
Control
Control+
HTL20 µM
HTL 200µM
Hcy 200µM
Hcy 200µM
N-Hcy 20 µM
N-Hcy 10 µM
(P)
+ miR-155 inhibitor
(Q)
+ miR-320c inhibitor
LC3
ATG7
ATG5
GAPDH
Control
Control+
HTL20 µM
HTL 200µM
Hcy 200µM
Hcy 200µM
N-Hcy 20 µM
N-Hcy 10 µM
GAPDH
Figure 6N, O, P, R - uncropped images
Control
Control+
HTL20 µM
Hcy 200 µM
HTL 200 µM
Hcy 200 µM
N-Hcy 20 µM
N-Hcy 10 µM
